# Isolation of Hv-CRKP with co-production of three carbapenemases (*bla*_KPC_, *bla*_OXA-181_ or _OXA-232_, and *bla*_NDM-1_) and a virulence plasmid: a study from a Chinese tertiary hospital

**DOI:** 10.3389/fmicb.2023.1182870

**Published:** 2023-05-24

**Authors:** Ping Li, Wan-ying Luo, Tian-Xin Xiang, Ting-xiu Peng, Shuai Luo, Zhi-yong He, Wenjian Liao, Dan-Dan Wei, Peng Liu, La-gen Wan, Wei Zhang, Yang Liu

**Affiliations:** ^1^Department of Pulmonary and Critical Care Medicine, The First Affiliated Hospital of Nanchang University, Nanchang University, Nanchang, China; ^2^Jiangxi Institute of Respiratory Disease, The First Affiliated Hospital of Nanchang University, Nanchang, China; ^3^Yichun People's Hospital, Yichun, China; ^4^Department of Infectious Diseases, The First Affiliated Hospital of Nanchang University, Nanchang University, Nanchang, China; ^5^Department of Clinical Laboratory, Medical Center of Burn Plastic and Wound Repair, The First Affiliated Hospital of Nanchang University, Nanchang University, Nanchang, China; ^6^National Regional Center for Respiratory Medicine, China-Japan Friendship Jiang Xi Hospital, Nanchang, Jiangxi, China

**Keywords:** *Klebsiella pneumoniae*, carbapenem-resistance, mobilized virulence factors, *bla*_OXA_ gene, antimicrobial-resistant genes

## Abstract

**Background:**

The worldwide dissemination of *K. pneumoniae* isolates is a significant public health concern, as these organisms possess a unique capacity to acquire genetic elements encoding both resistance and hypervirulence. This study aims to investigate the epidemiological, resistance, and virulence characteristics of *K. pneumoniae* isolates that carry both virulence plasmids and *bla*_OXA-48-like_ genes in a tertiary hospital in China.

**Methods:**

A total of 217 clinical isolates of carbapenem-resistant *K. pneumoniae* (CRKP) were collected between April 2020 and March 2022. The antimicrobial susceptibility test was conducted to evaluate the drug resistance profile. All isolates were screened for the presence of genes encoding carbapenemases (*bla*_KPC_, *bla*_NDM_, *bla*_IMP_, *bla*_VIM_, and *bla*_OXA-48-like_), ESBLs genes (*bla*_CTX-M_, *bla*_SHV_, *bla*_TEM_), and virulence plasmid pLVPK-borne genes (*rmpA*, *rmpA2*, *iucA*, *iroB*, and *peg344*) using polymerase chain reaction (PCR) amplification. Clonal lineages were assigned using multilocus sequence typing (MLST) and pulsed-field gel electrophoresis (PFGE). The plasmid incompatibility groups were identified using PCR-based replicon typing (PBRT). The transferability of carbapenemase-encoding plasmids and pLVPK-like virulence plasmids was assessed via conjugation. The plasmid location of *rmpA2* was determined using S1-Pulsed Field Gel Electrophoresis (S1-PFGE) and southern blotting hybridization. The virulence potential of the isolates was assessed using the string test, capsular serotyping, serum killing assay and a Galleria mellonella larval infection model.

**Results:**

Of the 217 CRKP clinical isolates collected, 23% were identified as carrying *bla*_OXA-48-like_ genes. All *bla*_OXA-48-like_ isolates exhibited resistance to commonly used clinical antimicrobial agents, except for ceftazidime/avibactam, colistin, tigecycline, trimethoprim-sulfamethOXAzole, polymyxin B, and nitrofurantoin. The main common OXA-48-like carbapenemase enzymes were found to be *bla*_OXA-181_ and *bla*_OXA-232_. MLST and PFGE fingerprinting analysis revealed clonal transmission and plasmid transmission. OXA-48-like producing CRKP isolates mainly clustered in K64 ST11 and K47 ST15. Results of the string Test, serum killing assay (*in vitro*) and *Galleria mellonella* infection model (*in vivo*) indicated hypervirulence. PBRT showed that the *bla*_OXA-181_ and *bla*_OXA-232_ producing hypervirulent carbapenem-resistant *Klebsiella pneumoniae* (Hv-CRKP) were mainly carried on ColE-type, IncF, and IncX3. Eight clinical isolates of hv-CRKP were identified as carrying three carbapenem-resistant genes (*bla*_KPC_, *bla*_OXA-181 or OXA-232_, and *bla*_NDM-1_). Moreover, Southern blotting hybridization revealed that all eight isolates had a pLVPK-like virulent plasmid (138.9–216.9 kb) with an uneven number and size of plasmid.

**Conclusion:**

In our investigation, we have observed the emergence of hv-CRKP carrying *bla*_OXA-48-like_ genes, which identified two genetic relationships: clonal transmission and plasmid transmission. PBRT analysis showed that these genes were mainly carried on ColE-type, IncF, and IncX3 plasmids. These isolates have been shown to be hypervirulent *in vitro* and *in vivo*. Additionally, eight clinical isolates of hv-CRKP were identified as carrying three carbapenem-resistant genes (*bla*_KPC_, *bla*_OXA-181 or OXA-232_, and *bla*_NDM-1_) and carrying a pLVPK-like virulent plasmid. Hence, our findings highlight the need for further investigation and active surveillance of hypervirulent OXA-48-like producing Hv-CRKP isolates to control their transmission.

## 1. Introduction

The Antimicrobial resistance (AMR) is a serious threat to global health, according to the World Health Organization. Among clinical pathogens, *K. pneumoniae* is particularly concerning due to its propensity to acquire multidrug resistance and hypervirulence-encoding mobile genetic elements (Yang et al., 2021). Carbapenem resistance in *K. pneumoniae* is often mediated by plasmid-encoded carbapenemase enzymes, such as *bla*_KPC_, *bla*_NDM_, and *bla*_OXA-48-like_ enzyme (Han et al., 2020).

Hypervirulent *K. pneumoniae*, first identified from cases of liver abscess, has been increasingly reported worldwide (Russo and Marr, 2019). A recent study demonstrated that *iroB*, *iucA*, *peg-344*, *rmpA*, and *rmpA2* were the most accurate molecular markers for defining hvKP, all of which have been shown to be located in the virulence plasmid (Russo et al., 2018). In recent years, more and more *K. pneumoniae* isolates integrating both hypervirulence and carbapenem resistance phenotypes have been identified, creating hypervirulent and carbapenem-resistant *K. pneumoniae* that result in devastating clinical outcomes (Yang et al., 2022).

Surveillance studies have revealed that OXA-48-like β-lactamases are among the 2nd or 3rd most common carbapenemases found in Enterobacterales globally (Pitout et al., 2019). OXA-48-like carbapenemases are mainly found in *K. pneumoniae* isolates submitted from hospital sites and have been increasing toward the end of surveillance periods (de Jonge et al., 2016; Karlowsky et al., 2017). Data from global surveillance programs such as SMART (Karlowsky et al., 2017) and INFORM (de Jonge et al., 2016) show that 27% of carbapenemase-producing Enterobacterales (CPE; *n* = 1,615) carry *bla*_OXA-48-like_ carbapenemases (compared to 55% *bla*_KPCs_ and 26% *bla*_NDMs_). In some regions, such as the Middle East, North Africa, and certain European countries like Belgium and Spain, OXA-48-like enzymes were the most prevalent carbapenemases among Enterobacterales (Pitout et al., 2019).

In recent years, cases of OXA-48-like *K. pneumoniae* isolates have been on the rise in China. For instance, OXA-232-producing CRKP was first isolated from five neonatal patients in China in 2017 (Yin et al., 2017), while the first report of OXA-181-producing *K. pneumoniae* from the fecal specimen of a patient in China was in 2020 (Liu et al., 2020). Subsequent reports have documented an increasing number of *bla*_OXA-48-like_ *K. pneumoniae* isolates in China (Liu et al., 2020; Shi et al., 2020; Jia et al., 2021). In December 2016, the draft genome sequences of three hypervirulent CRKP isolates from India were reported to harbor *bla*_OXA_ genes (*bla*_OXA-232_, *bla*_OXA-181_, and *bla*_OXA-1_) along with the *rmpA2* gene (Shankar et al., 2016). While China reported the emergence of OXA-232 carbapenemase-producing *K. pneumoniae* carrying a pLVPK-like virulence plasmid among elderly patients in February 2019, these isolates were not hypervirulent despite carrying a virulence plasmid (Shu et al., 2019). This study aims to investigate the resistance mechanisms and molecular epidemiology of hypervirulent *Klebsiella pneumoniae* isolates producing OXA-48-like carbapenemases in a Chinese tertiary hospital.

## 2. Materials and methods

### 2.1. Bacterial isolates and definitions

Between April 2020 and March 2022, the First Affiliated Hospital of Nanchang University in China collected 217 unique clinical carbapenem-resistant *K. pneumoniae* isolates, characterized by minimum inhibitory concentrations (MICs) of ertapenem >0.5 μg/mL, imipenem >4 μg/mL or meropenem >8 μg/mL. All isolates were identified using the VITEK 2 automated system (bioMerieux, Marcy l’Etoile, France) and the MALDI-TOF MS system (Bruker Daltonics, Billerica, MA, United States) and stored at −80°C until use. The MIC of tigecycline was determined through the *E*-test (AB Biodisk, Solna, Sweden) on Mueller-Hinton media. Susceptibility to colistin and tigecycline was determined according to the European Committee on Antimicrobial Susceptibility Testing (EUCAST) guidelines,^1^ while susceptibilities to other agents were interpreted using the Clinical and Laboratory Standards Institute (CLSI) breakpoints (document M100-S32).

All isolates were screened for the presence of genes encoding carbapenemases (*bla*_KPC_, *bla*_NDM_, *bla*_IMP_, *bla*_VIM_, and *bla*_OXA-48-like_), ESBLs genes (*bla*_CTX-M_, *bla*_SHV_, *bla*_TEM_), and virulence plasmid pLVPK-borne genes (*rmpA*, *rmpA2*, *iucA*, *iroB*, and *peg344*) using polymerase chain reaction (PCR) amplification, as previously described (Liu et al., 2019). PCR products were visualized by agarose gel electrophoresis and sequencing, and the sequence analysis of PCR products was conducted by Sangon Biotech (Shanghai, China) and aligned in *bla*_ST_ searches in the NCBI Genbank. Isolates positive for *bla*_OXA-48-like_ genes and virulence genes were further studied.

### 2.2. Clinical data collection

The clinical data used in this study were obtained from the Electronic Medical Records of inpatients at the First Affiliated Hospital of Nanchang University. The data included patient demographics, date of isolation, clinical diagnosis, specimens, ward admission, antimicrobial treatment, and hospitalization outcomes. The study and consent procedures were approved by the Ethical Committee of the First Affiliated Hospital of Nanchang University.

### 2.3. Molecular epidemiology analysis: multilocus sequence typing and pulsed-field gel electrophoresis

MLST and PFGE was used to evaluate the genetic relatedness of isolates positive for *bla*_OXA-48-like_ genes and virulence genes.

MLST was conducted in accordance with the protocol outlined on the Pasteur Institute MLST website, using seven conserved housekeeping genes (gapA, infB, mdh, pgi, phoE, rpoB, and tonB). The resulting MLST amplicons were purified and sequenced by Sangon Biotech in Shanghai, China, and compared to those in the MLST database to determine the sequence type (ST).

PFGE using XbaI from TaKaRa was performed. DNA fragments were then separated via a CHEF DR III apparatus (Bio-Rad, Richmond, CA, United States), with Salmonella serotype Braenderup isolate H9812 serving as a molecular marker. Subsequently, BioNumerics software version 7.6 was utilized to construct a tree diagram using the unweighted Pair-Group Method with Arithmetic means (UPGMA) and the Dice similarity coefficient (SD) with a 1.5% position tolerance. Isolates were considered genetically similar if their Dice coefficient correlation exceeded 80%, in line with the “possibly related (4–6 bands difference)” criteria developed by Tenover et al. (1995).

### 2.4. Plasmid analyses

#### 2.4.1. Plasmid conjugation

Conjugation was employed to evaluate the transferability of plasmids carrying carbapenemases (*bla*_KPC_, *bla*_OXA-181 or OXA-232_, and *bla*_NDM-1_) and pLVPK-like virulence plasmid. Eight clinical isolates of CR-hvKP carrying carbapenem-resistant genes (*bla*_KPC_, *bla*_OXA-181 or OXA-232_, and *bla*_NDM-1_) were used as donors, while rifampicin-resistant *E. coli* EC600 was used as the recipient. Both donor and recipient isolates were cultured in Luria-Bertani broth (10 g/L tryptone, 5 g/L yeast extract, 5 g/L NaCl) at 37°C with shaking (180 rpm) until they reached their exponential growth phase (OD600 = 0.4–0.6). The overnight cultures were then mixed in a 1:1 ratio and incubated at 37°C for 16–20 h. After incubation, 100 μL of the sample was spread onto MH agar plates containing imipenem (5 μg/mL), potassium tellurite (5 μg/mL), and rifampicin (600 μg/mL).

#### 2.4.2. PCR-based replicon typing (PBRT)

Plasmid incompatibility groups were determined using PBRTas previously described in literature (Carattoli et al., 2005; Carattoli, 2009; Carattoli, 2011). PBRT was used to tract the plasmids conferring drug resistance in epidemiological of transconjugants and isolates positive for blaOXA-48-like genes and virulence genes. The identified plasmid incompatibility groups included HI1, HI1b, HI2, I1-γ, L/M, N, FIA, FIB, FIC, FIIA, F, K, B/O, W, Y, P, A/C, T, X, X1, X2, X3, and X4.

#### 2.4.3. S1-pulsed field gel electrophoresis and southern blotting hybridization

Plasmid characteristics were assessed by S1-PFGE. Southern blotting hybridization was performed to determine the plasmid location of the virulence plasmid with a *rmpA2* gene. In brief, the isolates were embedded in 1% Seakem Gold agarose and digested with S1-nuclease (Takara, Otsu, Japan) at 37°C for 30 min, and plasmids were separated on a CHEF DR III apparatus (Bio-Rad, Richmond, CA, United States) for 18 h at 14°C, using a 0.8% agarose gel and run conditions of 6 V/cm and pulse times ranging from 2.16 s to 63.8 s. Plasmid molecular mass standards covering a range from 20.5 kb to 1,135 kb, isolated from Salmonella serotype Braenderup isolate H9812, were used. The transferred plasmids on the S1-PFGE gel were transferred to Hybond-N+ membranes (Amersham), following a previously described protocol (Liu et al., 2017). The probe labeling for *rmpA2* and hybridization were conducted using the DIG-High Prime DNA Labeling and Detection Starter Kit I, following the manufacturer’s instructions (CAT.NO.11745832910, Roche, Mannheim, Germany).

### 2.5. Virulence assessment of transformant

#### 2.5.1. Hyperviscous phenotype detection (string test)

For isolates that were positive for all the aforementioned virulence genes, hypermucoviscosity was defined as present when the viscous string was longer than 5 mm when colonies were stretched on an agar plate.

#### 2.5.2. Serum killing assay

In addition, we performed a serum killing assay to determine *in vitro* virulence, as described in previous literature (Liu et al., 2017). Briefly, serum was collected from healthy individuals and stored at −80°C. An inoculum of 106 CFU mid-log phase bacteria was incubated with 75% pooled human serum, and viable counts were recorded at 0, 1, 2, and 3 h of incubation at 37°C and 200 rpm. Each isolate was tested at least three times. The reaction to serum killing was classified into six grades and categorized as highly sensitive (grade 1 or 2), intermediately sensitive (grade 3 or 4), or resistant (grade 5 or 6). Grade 1 indicated viable counts <10% of the inoculum after 1 and 2 h, and < 0.1% after 3 h. Grade 2 referred to viable counts between 10 and 100% of the inoculum after 1 h and < 10% after 3 h. Grade 3 indicated viable counts exceeding those of the inoculum after 1 h but <100% after 2 and 3 h. Grade 4 referred to viable counts >100% of the inoculum after both 1 and 2 h but <100% after 3 h. Grade 5 referred to viable counts >100% of the inoculum at 1, 2, and 3 h, which decreased during the third hour. Grade 6 referred to viable counts that exceeded those of the inoculum at 1, 2, and 3 h and increased throughout this period. Isolates K. pneumonia ATCC 700603 and the hvKP isolates NTUH-K2044 were used as negative and positive controls, respectively, with serum killing sensitivity of grade 2 and resistance of grade 5.

#### 2.5.3. *Galleria mellonella* infection model

The larvae of *Galleria mellonella* (Gm) was an infection model for the virulent to evaluate study virulence of gram-negative bacteria isolates (Ennis and Sells, 1968; Asai et al., 2022), so we evaluated *in vivo* virulence using the *Galleria mellonella* infection model to assess hypervirulence. Microbial virulence in the *G. mellonella* infection model is typically assessed within 5 d and the most commonly used end point is the survival rate at different time points (Asai et al., 2022). Specific experimental steps was as previously described (Mclaughlin et al., 2014). In brief, 10 pathogen-free *G. mellonella* larvae weighing between 250 and 350 mg (purchased from Tianjin Huiyude Biotech Company, Tianjin, China) were used for each isolate. A mid-log-phase culture was washed and diluted with PBS, and each larva was inoculated by injecting 1 × 10^6^ CFU in a 10 ul aliquot into the hemocoel via the rear left pro leg. Survival rate was recorded every 24 h for 4 days, and larvae were kept in petri dishes at 37°C in the dark. All experiments were conducted in triplicate. The calculation of the LD50 value has been proposed to define hypervirulence in the *Galleria mellonella* infection model for *K. pneumoniae* isolates (Li et al., 2020). The HvKP isolate NTUH-K2044 and PBS were used as controls for high and low virulence, respectively. Statistical analyses were performed and visualized using GraphPad Prism 8.0.

### 2.6. Statistical analyses

The statistical analysis was performed using SPSS version 17.0 (SPSS, Chicago, IL, United States). Categorical variables were compared using either the chi-square test or Fisher’s exact test, and a *p*-value of less than 0.05 was considered statistically significant.

## 3. Results

### 3.1. Prevalence of CRKP co-carrying pLVPK-like virulence plasmid and *bla*_OXA-48-like_ carbapenemases genes in a Chinese tertiary hospital

**Figure 1:**
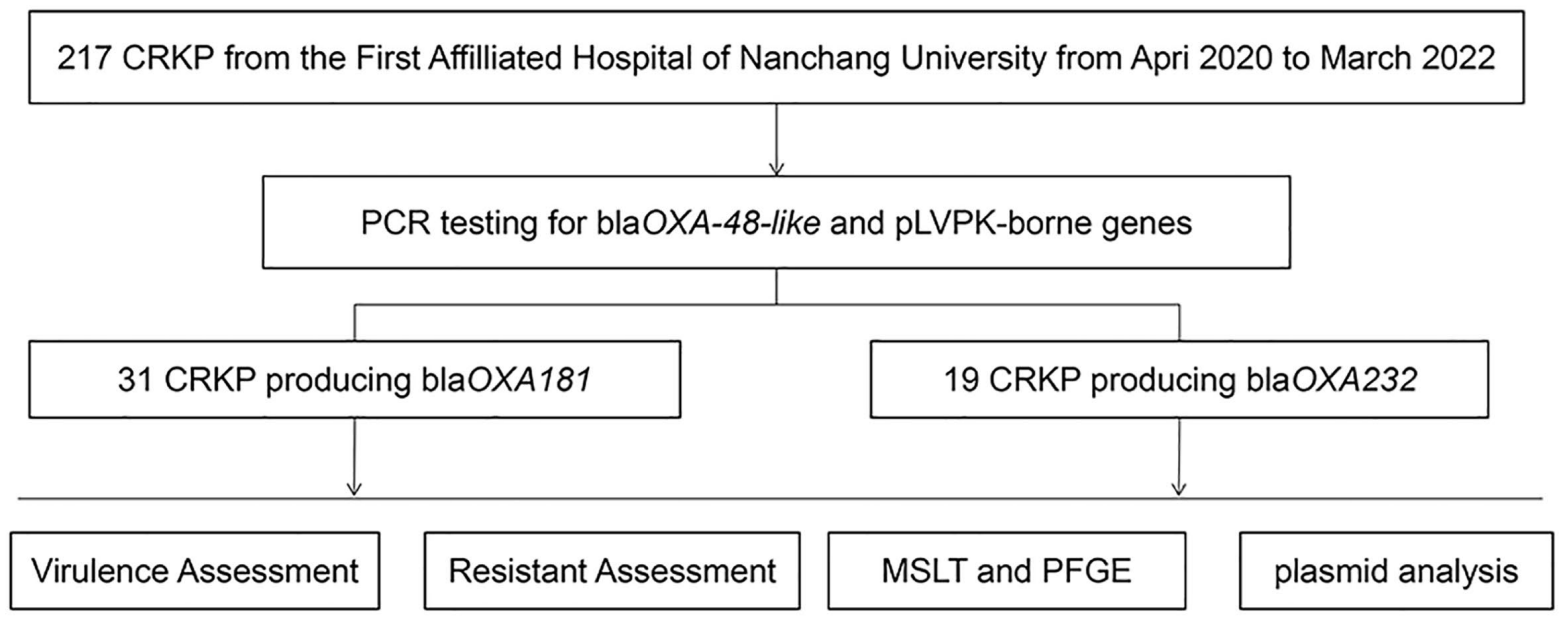
Schematic procedure of the study.

A total of 217 clinical isolates of CRKP were collected from our hospital between April 2020 and March 2022. Among these isolates, 50 (23%) carried both the pLVPK-like virulence plasmid and *bla*_OXA-48-like_ carbapenemase genes (Figure 1). These clinical isolates were obtained from various clinical specimens, including blood (Pitout et al., 2019), pus (Li et al., 2020), sputum (Khan et al., 2019), and urine (Asai et al., 2022) (Table 1). The ICU occupancy rate for these patients was 62% (31/50), and the overall mortality rate among inpatients involved in the outbreak was 52% (26/50). The median age of patients was 54.7 ± 12.6 years, and the male-to-female ratio was 2.3 (Table 1).

**Table 1:** The clinical data of patients infected with *K. pneumoniae* isolates co-carrying pLVPK virulence plasmid and blaOXA-48-like carbapenemases genes.

Patients	Isolates	Age	Gender	Date	Ward	Diagnosis	Specimens	Antimicrobial therapy	Outcome
patient01	oxakp01	58	Male	30-Jun-21	Department of Medical Rehabilitation	Intracerebral hemorrhage	Sputum	Cefoperazone/Sulbactam, Tigecycline	Recovered
patient02	oxakp02	51	Male	30-Sep-20	ICU	Respiratory failure	Sputum	Tigecycline, polymyxin B, Meropenem, Cefoperazone/Sulbactam	Recovered
patient03	oxakp03	54	Female	16-Nov-20	Department of Gastroenterology	Acute respiratory failure	Blood	–	Died
patient04	oxakp04	62	Female	19-Dec-20	Department of Neurosurgery	Hypoxic–ischemic encephalopathy	Blood	–	Recovered
patient05	oxakp05	62	female	19-Dec-20	Department of Neurosurgery	Gallstone with cholecystitis	Blood	Amikacin, Levofloxacin, Cefoperazone/Sulbactam, Imipenem	Recovered
patient06	oxakp06	40	Male	29-Oct-20	Department of Gastroenterology	Brain hernia and ventricular hemorrhage	Blood	Cefoperazone/Sulbactam, Tigecycline, Meropenem, Ceftazidime-avibactam	Died
patient07	oxakp07	57	Male	20-Sep-20	Department of Neurosurgery	Severe pneumonia	Sputum	–	Died
patient08	oxakp08	55	Male	28-Oct-20	Department of Neurosurgery	Septicemia and severe pneumonia	Sputum	Biapenem, Daltomycin, Tigecycline, Imipenem	Died
patient09	oxakp09	65	Male	28-Nov-20	Department of Respiration	Epidural hematoma	Sputum	Meropenem, Tigecycline, polymyxin B, Ceftazidime-avibactam, Amikacin	Recovered
patient10	oxakp10	40	Male	8-Nov-20	Department of Emergency Room	Acute severe pancreatitis, sepsis	Drainage	Tigecycline, Cefoperazone/Sulbactam, polymyxin B, Teicoplanin, Ceftazidime-avibactam	Died
patient11	oxakp11	70	Female	28-Nov-20	Department of Emergency Room	Multiple damages	Sputum	Tigecycline, Linezolid	Recovered
patient12	oxakp12	50	Male	7-Aug-21	ICU	Acute severe pancreatitis	Blood	Biapenem, Daltomycin, Tigecycline	Died
patient13	oxakp13	69	Male	20-Nov-20	Department of Neurosurgery	Acute pancreatitis	Sputum	Imipenem, Linezolid, Ceftazidime-avibactam	Died
patient14	oxakp14	68	Male	4-Dec-20	Department of Infectious Disease	Benign neoplasm of craniopharyngeal duct	Blood	Ceftazidime, Amikacin, Tigecycline, Ceftazidime-avibactam, polymyxin B, Cefoperazone/Sulbactam	Recovered
patient15	oxakp15	68	Male	4-Dec-20	Department of Infectious Disease	Acute severe pancreatitis	Blood	Biapenem, Tigecycline, polymyxin B, Teicoplanin	Died
patient16	oxakp16	24	Female	4-Nov-20	Department of Orthopedics	Lung adenocarcinoma and tracheal stent implantation	Urine	Biapenem	Recovered
patient17	oxakp17	87	Female	30-Nov-20	Department of General Practice	Acute severe pancreatitis	Blood	Biapenem, Daltomycin, Tigecycline	Died
patient18	oxakp18	50	Male	14-Aug-21	ICU	Cervical dislocation	Blood	Amikacin, polymyxin B, Cefoperazone/Sulbactam	Recovered
patient19	oxakp19	44	Female	16-Sep-21	ICU	Severe pneumonia	Blood	Imipenem, Tigecycline, Amikacin, polymyxin B, Meropenem, Ceftazidime-avibactam	Died
patient20	oxakp20	43	Male	3-Oct-21	Department of Respiration	Multiple organ failure	Blood	Ceftazidime-avibactam, polymyxin B, Meropenem	Recovered
patient21	oxakp21	43	Male	5-Oct-21	Department of Respiration	Atlas fracture	Blood	Tigecycline, Cefoperazone/Sulbactam, polymyxin B, Teicoplanin, Meropenem	Recovered
patient22	oxakp22	37	Female	24-Nov-22	ICU	Cerebral contusion	Blood	SMZ, Biapenem, Teicoplanin, Tigecycline, Meropenem, Piperacillin-tazobactam	Recovered
patient23	oxakp23	37	Female	24-Nov-21	ICU	Intracerebral hemorrhage	Blood	Linezolid, Meropenem	Died
patient24	oxakp24	50	Female	21-Nov-22	Department of Respiration	Explosive myocarditis	Sputum	Daltomycin, polymyxin B	Died
patient25	oxakp25	37	Female	10-Nov-21	ICU	Spinal injuries	Drainage	polymyxin B	Recovered
patient26	oxakp26	65	Male	1-Nov-21	Department of Respiration	Basal ganglia hemorrhage	Pus	Linezolid, Meropenem, polymyxin B	Recovered
patient27	oxakp27	62	Male	3-Dec-22	Department of Orthopedics	Acute severe pancreatitis and sepsis	Blood	Tigecycline, Cefoperazone/Sulbactam, polymyxin B, Teicoplanin, Ceftazidime-avibactam	Died
patient28	oxakp28	31	Male	9-Dec-21	Department of Medical Rehabilitation	Very severe open craniocerebral injury	Urine	Linezolid, polymyxin B, Teicoplanin	Died
patient29	oxakp29	64	Male	3-Dec-21	Department of General Surgery	Benign neoplasm of pituitary gland	Drainage	Linezolid, Tigecycline, Levofloxacin	Recovered
patient30	oxakp30	46	Male	7-Dec-21	ICU	Benign neoplasm of pituitary gland	Pus	Linezolid, Tigecycline, Levofloxacin	Recovered
patient31	oxakp31	51	Male	2-Jan-22	Department of Emergency Room	Chronic liver failure	Blood	Imipenem	Died
patient32	oxakp32	66	Male	6-Jan-22	Department of Neurosurgery	Brain stem neoplasms	Blood	Ceftazidime, Amikacin, Tigecycline	Died
patient33	oxakp33	46	Male	3-Jan-22	ICU	Chronic liver failure	Pus	Imipenem, Linezolid	Died
patient34	oxakp34	50	Female	3-Jan-22	Department of General Surgery	Esophageal cancer surgery	Drainage	Piperacillin-tazobactam钠, Meropenem	Recovered
patient35	oxakp35	71	Male	26-Jan-22	Department of Respiration	Common bile duct stone with cholecystitis	Sputum	Imipenem, Linezolid	Recovered
patient36	oxakp36	68	Male	2-Feb-22	ICU	Cerebral contusion	Sputum	Imipenem, Cefoperazone/Sulbactam, Tigecycline	Recovered
patient37	oxakp37	60	Female	18-Feb-22	ICU	Thalamic hemorrhage	Blood	polymyxin B, Linezolid	Died
patient38	oxakp38	67	Male	18-Feb-22	Department of Infectious Disease	Septic shock	Drainage	Meropenem, polymyxin B	Died
patient39	oxakp39	65	Male	19-Feb-22	Department of General Surgery	Adult Ph acute lymphoblastic leukemia	Drainage	Ceftazidime-avibactam, polymyxin B, Tigecycline, Teicoplanin	Died
patient40	oxakp40	52	Male	25-Feb-22	Department of Neurosurgery	Hepatapostema	Blood	Ceftazidime-avibactam, Tigecycline, Imipenem, Amikacin	Died
patient41	oxakp41	66	Female	27-Feb-22	Department of Emergency Room	Biliary tract infection	Drainage	Cefoperazone/Sulbactam	Recovered
patient42	oxakp42	65	Male	6-Mar-22	Department of General Surgery	Hepatapostema	Wound	–	Died
patient43	oxakp43	55	Male	5-Mar-22	ICU	Hepatapostema	Blood	Biapenem, Tigecycline	Recovered
patient44	oxakp44	57	Male	7-Mar-22	ICU	Hepatapostema	Wound	polymyxin B, Ceftazidime-avibactam	Died
patient45	oxakp45	40	Male	22-Feb-22	Department of Emergency Room	Acute pancreatitis	Sputum	Biapenem, Teicoplanin	Recovered
patient46	oxakp46	57	Male	8-Mar-22	ICU	Paraplegia	Drainage	Amikacin	Recovered
patient47	oxakp47	53	Male	10-Mar-22	Department of Neurosurgery	Biliary tract infection	Drainage	Amikacin, Cefoperazone/Sulbactam	Recovered
patient48	oxakp48	55	Male	11-Mar-22	ICU	Thalamic hemorrhage	Sputum	polymyxin B, Linezolid	Died
patient49	oxakp49	70	Male	14-Mar-22	ICU	Idiopathic thrombocytopenic purpura	Sputum	Meropenem, Teicoplanin	Died
patient50	oxakp50	35	Female	13-Mar-22	ICU	Septic shock	Blood	Meropenem, polymyxin B	Died

**Figure 2:**
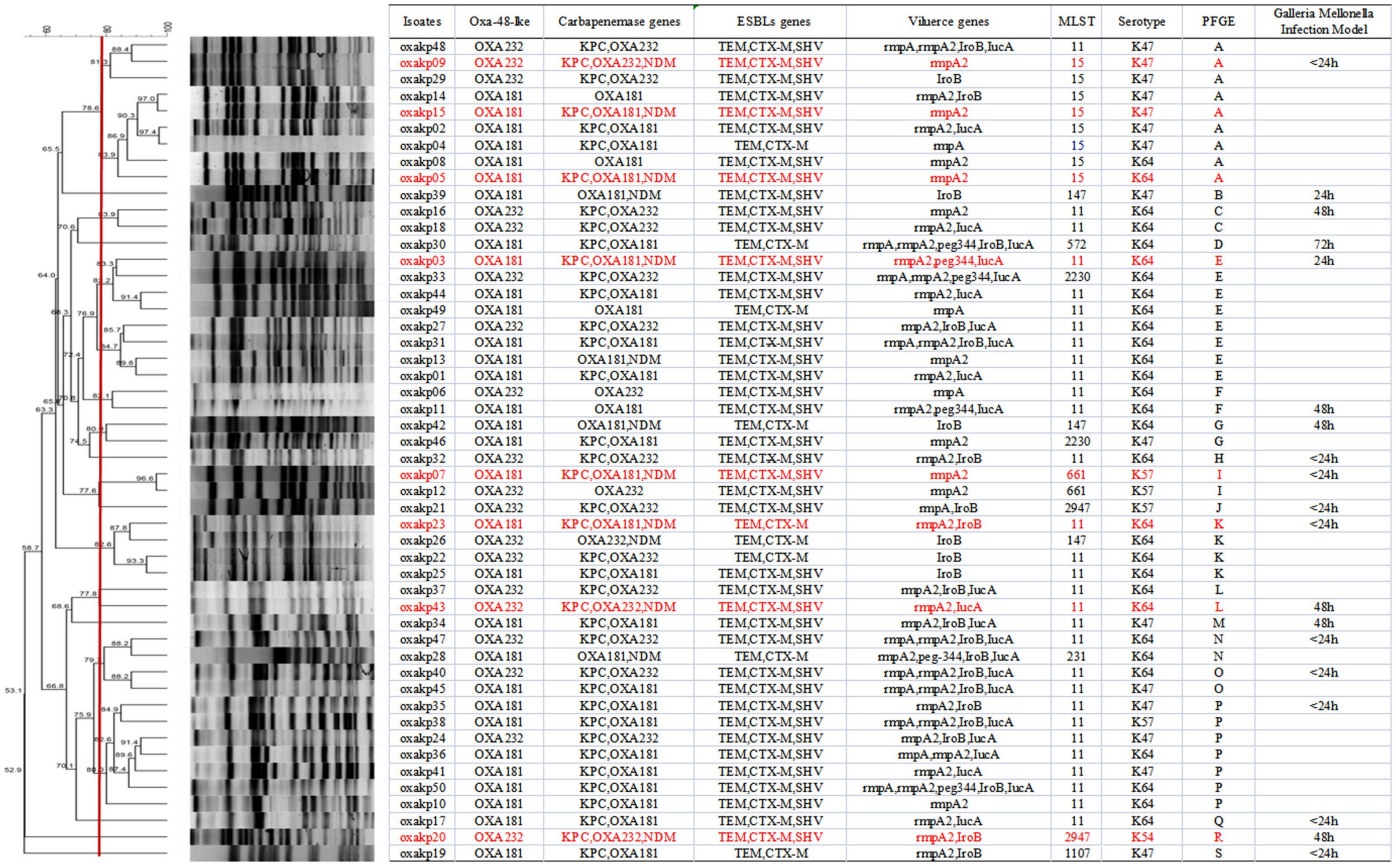
PFGE typing of 50 clinical OXA-48-like positive CRKP isolates. Genomic DNA from each research strains was digested using Xba I and the digests were subjected to PFGE to generate diagnostic genomic DNA fragmentation fingerprints. The dendrogram of the PFGE profiles was clustered by the UPGAMA on the basis of the Dice similarity by the bionumber software. The red line delineates 80% of the boundary. The strains producing carbapenemases (kpc, OXA, and NDM) are indicated in red font.

Upon sequence comparison with GenBank, we found that OXA-181 (62%, 31/50) and OXA-232 (38%, 19/50) were the most common carbapenemases identified among the OXA-48-like carbapenemases. All the isolates were found to be positive for the presence of *bla*_CTX-M_ and *bla*_TEM_ genes. Additionally, the *bla*_SHV_ gene was detected in over 80% of the isolates. As shown in Figure 2, eight clinical isolates of CRKP carried three carbapenem-resistant genes, including five isolates producing *bla*_KPC + NDM + OXA-181_ and three isolates producing *bla*_KPC + NDM + OXA-232_.

### 3.2. Molecular characteristics

MLST analysis of 50 isolates of OXA-48-like producing CRKP identified nine distinct sequence types (STs), as demonstrated in Figure 2. The most frequently encountered ST was ST11, which accounted for 30 out of 50 isolates, followed by ST15, which was found in eight isolates. No notable differences were observed in the STs of isolates carrying *bla*_OXA-181_ versus those carrying *bla*_OXA-232_. PFGE analysis demonstrated that CRKP isolates producing both *bla*_OXA-181_ and *bla*_OXA-232_ displayed 19 distinct PFGE patterns, respectively, as depicted in Figure 2. Notably, Cluster A, E, and P exhibited clonal relatedness. Furthermore, both clonal and plasmid transmission was observed based on PFGE analysis. The combined results of PFGE and MLST analysis showed that CRKP isolates co-carrying pLVPK-like virulence plasmid and *bla*_OXA-181 and OXA-232_ resistant plasmid mainly clustered in ST11 and ST15 isolates.

### 3.3. Resistant assessment of *Klebsiella pneumoniae* clinical isolates co-carrying pLVPK-like virulence plasmid and *bla*_OXA-181 and OXA-232_ resistant genes

**Figure 3:**
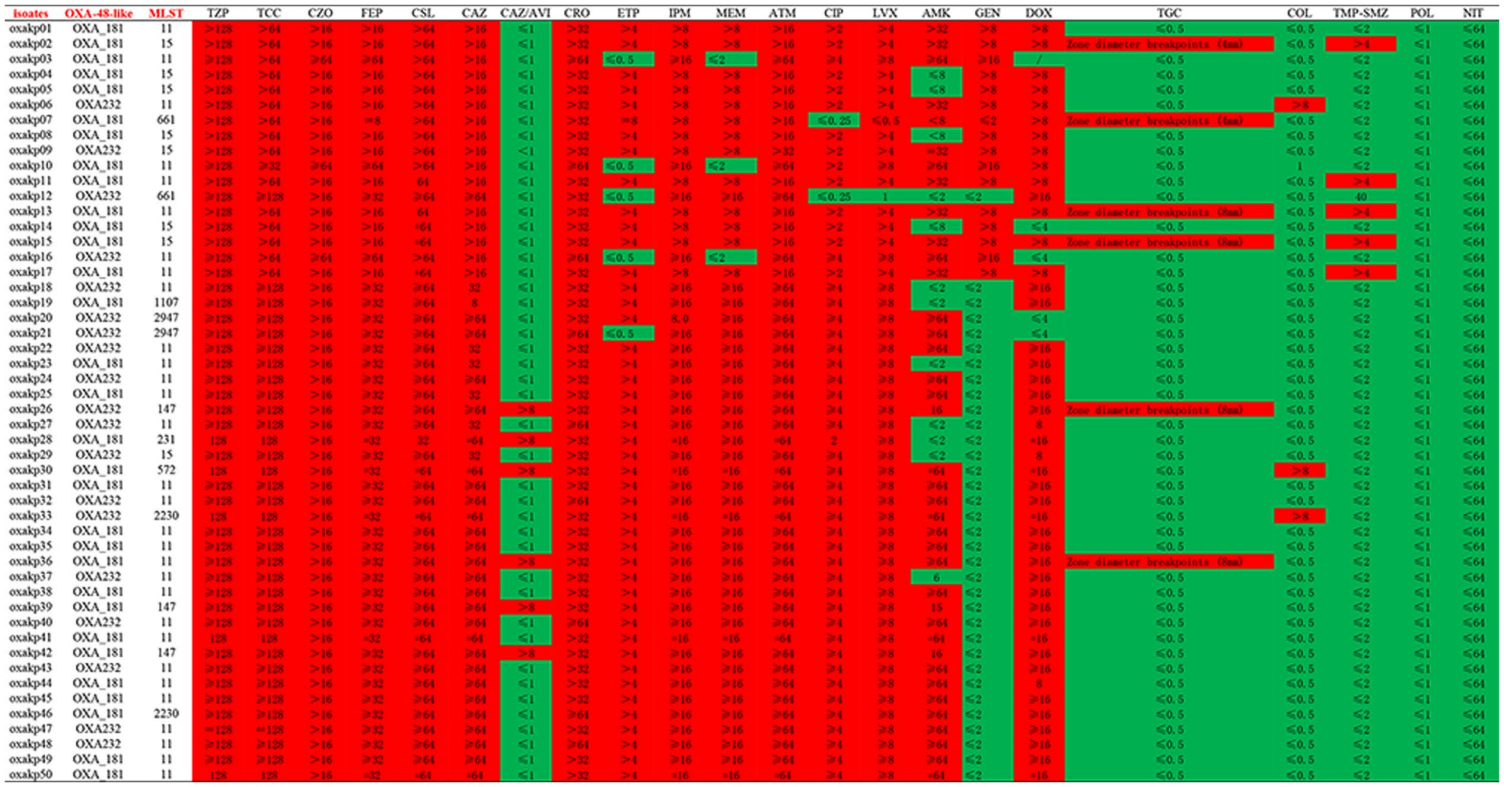
*In vitro* activities of antimicrobial agents and disinfectants against CRKP isolates co-carrying pLVPK virulence plasmid and bla*OXA-48-like* carbapenemases genes.

Figure 3 present the antibacterial susceptibility of 50 OXA-48-like producing *K. pneumoniae* isolates. All isolates exhibited resistance to commonly used clinical antimicrobial agents, except for ceftazidime/avibactam, colistin, tigecycline, trimethoprim-sulfamethOXAzole, polymyxin B, and nitrofurantoin. Specifically, the clinical isolates in this study demonstrated complete resistance to Piperacillin-tazobactam, Ticarcillin-clavulanic acid, Cefazolin, Cefepime, Cefoperazone/Sulbactam, Ceftazidime, Ceftriaxone, Aztreonam, and Imipenem (100%). The rates of antibacterial resistance to LevoflOXAcin, CiproflOXAcin, Meropenem, Ertapenem, and Doxycycline were 96, 96, 94, 90, and 90%, respectively, with 48/50, 48/50, 47/50, 45/50, and 45/50 isolates exhibiting resistance to each drug, respectively. Furthermore, these isolates were fully sensitive to Polymixin B and Nitrofurantoin. The tigecycline and colistin MICs were each <1 μg/mL, except for six isolates that had a TGC zone diameter of 4, 4, 8, 8, 8, and 8 mm.

Figure 2 illustrates that all these 50 CRKP carried at least one carbapenemase genes (*bla*_KPC_, *bla*_NDM_, *bla*_OXA_) or ESBL genes (*bla*_CTX-M_, *bla*_SHV_, *bla*_TEM_). As depicted in Figure 2, our study identified eight clinical isolates of Hv-CRKP that carried three carbapenem-resistant genes, namely five of *bla*_KPC + OXA-181 + NDM-1_ and three of *bla*_KPC + OXA-233 + NDM-1_. To our knowledge, this is the first report of the co-production of three carbapenemase genes (*bla*_KPC + NDM + OXA181 or OXA232_) and the pLVPK-like virulence plasmid in CRKP isolates.

**Figure 4:**
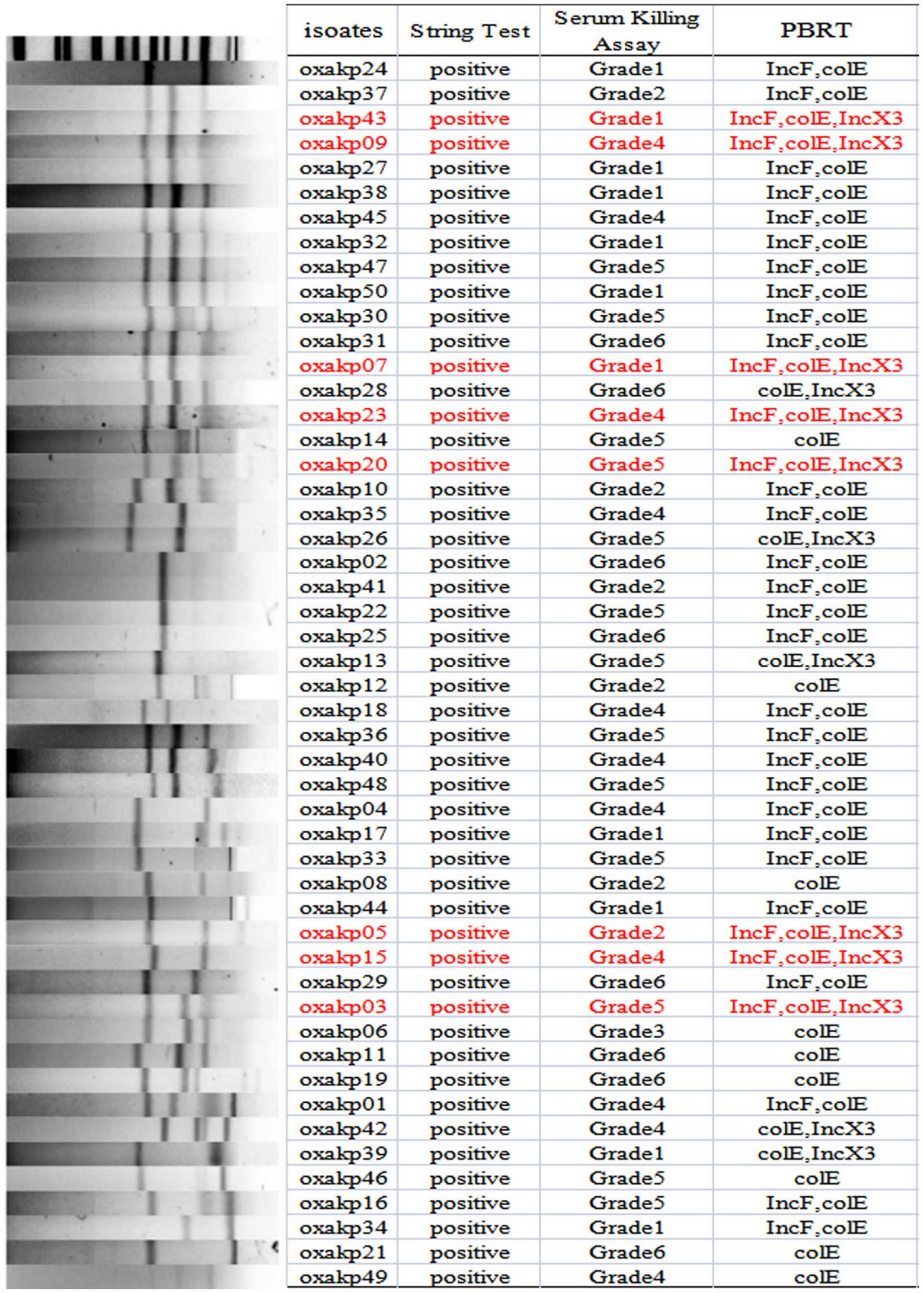
S1-PFGE typing of 50 clinical OXA-48-like Positive *K. pneumoniae* isolates. Genomic DNA from each research strains was digested using S1 and the digests were subjected to DNA fragments were separated with a CHEF DR III apparatus.

The plasmid-borne resistance to *bla*_OXA-181 and OXA-232_ producing CRKP was mainly attributed to ColE-type plasmids (100%, 50/50), IncF plasmids (72%, 36/50), and IncX3 plasmids (26%, 13/50), with IncX3 plasmids always associated with *bla*_NDM_ (Figure 4). To evaluate the transferability of these resistant plasmids, we selected the aforementioned eight isolates that carry three carbapenemase resistance genes and performed the plasmid conjugation experiment. Fortunately, all eight bacterial isolates were successfully conjugated.

### 3.4. Virulence assessment of *Klebsiella pneumoniae* clinical isolates co-carrying p-LVPK-like virulence plasmid and *bla*_OXA-181 and OXA-232_ resistant genes

As showed in Figure 2, it was observed that all *bla*_OXA-48-like_ positive CR-hvKP isolates harbored at least one virulence gene located on a pLVPK-like virulence plasmid (including *iroB*, *iucA*, *peg-344*, *rmpA*, and *rmpA2* genes). The string test was positive for all these isolates. Capsular serotyping showed that 30 isolates were K64, 15 were K47, 4 were K57, and 1 was K54, while K1 and K2 types were not detected. No significant difference was observed between the blaOXA-181 and blaOXA-232 groups. Our findings suggest that *K. pneumoniae* isolates co-carrying p-LVPK-like virulence plasmids and *bla*_OXA-181 and OXA-232_ resistant plasmids are mainly clustered in K64 and K47 isolates.

**Figure 5:**
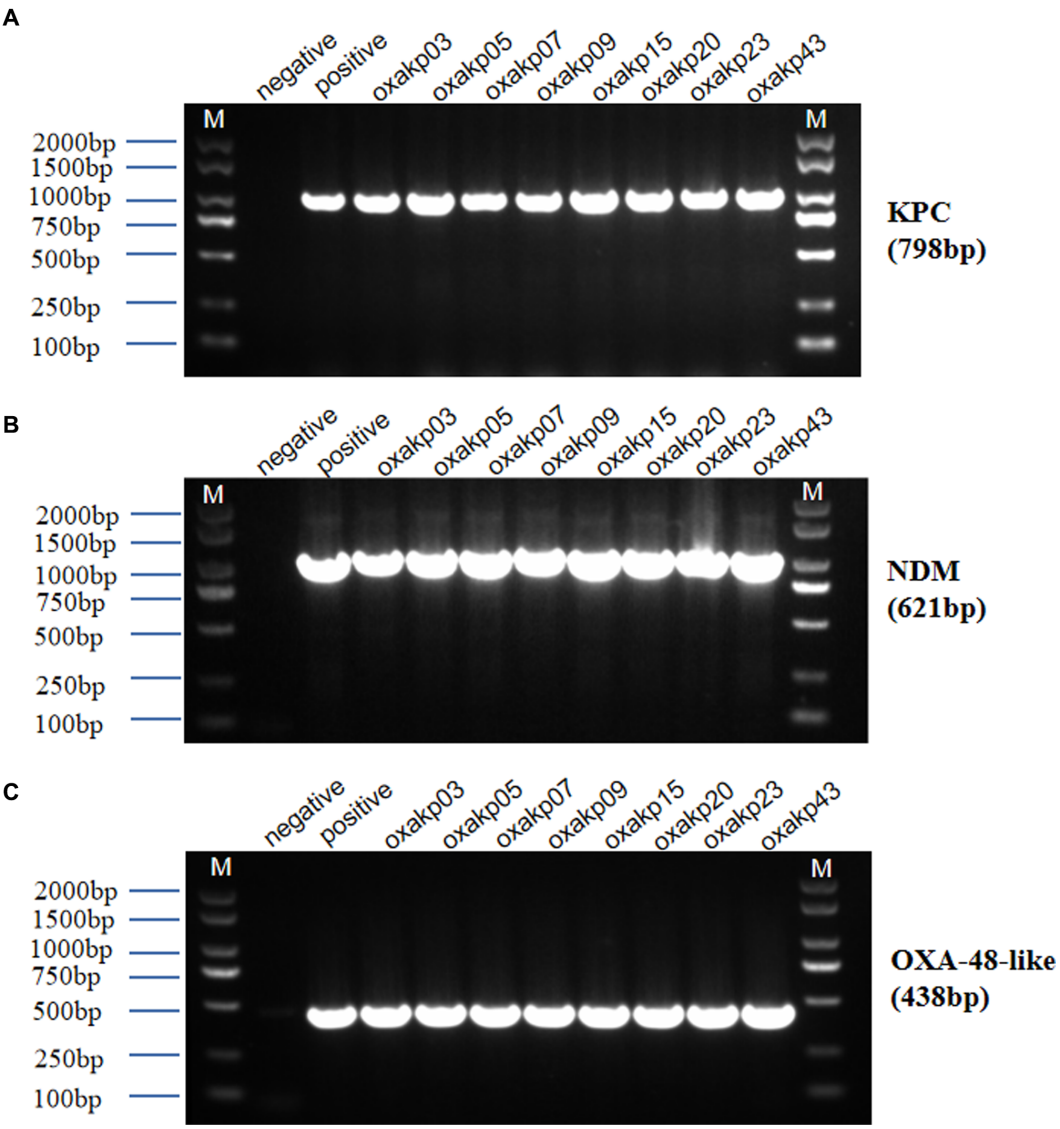
Agarose gel electrophoresis was performed for eight clinical isolates of Hv-CRKP carrying three carbapenem-resistant genes **(A–C)**. The gel showed PCR products of expected lengths for the *bla*_KPC_ gene (approximately 798 bp), *bla*_NDM_ gene (approximately 621 bp), and *bla*_OXA-48-like_ gene (approximately 438 bp). M:2000 bp size marker, Negative Control did not show amplification.

**Figure 6:**
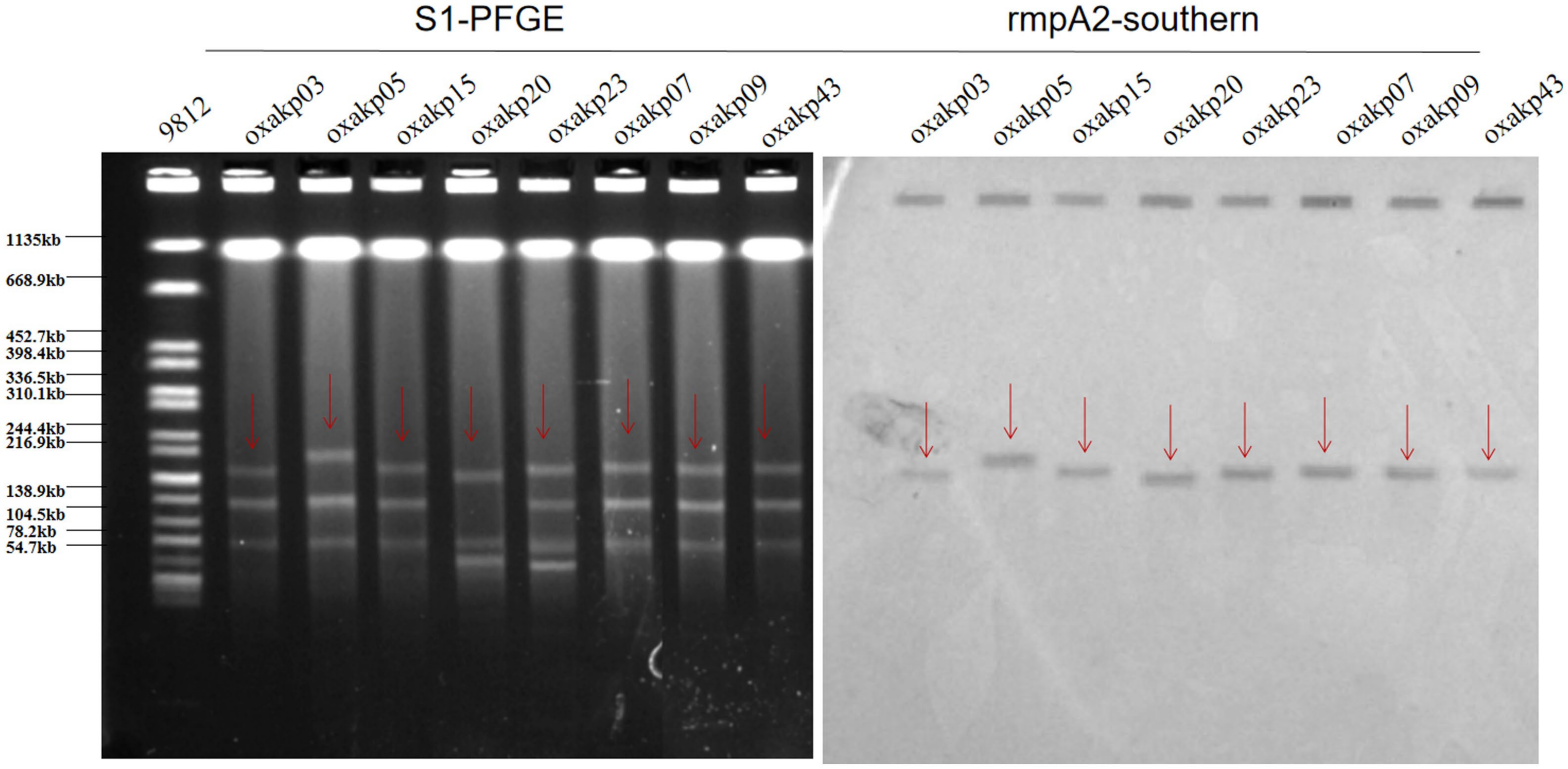
S1-PFGE patterns and rmpA2-southern of the eight hv-CRKP strains co-producing three carbapenemases harbored a pLVPK-like virulent plasmid (ranging from 138.9 to 216.9 kb).

The presence of hypermucoviscosity and plasmid-borne genes resembling pLVPK in the isolated strains suggests hypervirulence. *In vitro* experiments confirmed that the strains exhibited serum resistance, with a survival rate of approximately 78% after 60 min of incubation with serum (Figure 4). To assess virulence *in vivo*, one isolate was randomly selected from each typing cluster based on PFGE. The results, shown in Figure 2, indicate that 19 CRKP isolates were chosen. When a 10^6^ CFU suspension of the isolates was used to infect *Galleria mellonella* larvae, 18 isolates had a survival rate of less than 48 h, which was similar to that of a virulent strain of NUTH-K2044 (Figure 2). Eight CRKP isolates, which co-produced three carbapenemases (*bla*_KPC_, *bla*_OXA-181_ or _OXA-232_, and *bla*_NDM-1_) (Figure 5), were found to harbor a pLVPK-like virulent plasmid (ranging from 138.9 to 216.9 kb) as determined by Southern blotting hybridization of the virulence gene *rmpA2* (Figure 6).

## 4. Discussion

Bacteria carrying *bla*_OXA-48_, *bla*_OXA-181_, and *bla*_OXA-232_ are emerging globally, particularly in *K. pneumoniae* and *E. coli*, but their incidence is likely underreported due to laboratory detection challenges (Pitout et al., 2019). The rapid emergence and spread of multidrug-resistant (MDR) and hypervirulent *K. pneumoniae* isolates is a growing concern in clinical settings (Zhang et al., 2020). In recent years, two evolutionary pathways in *K. pneumoniae* have converged, leading to the emergence of carbapenem-resistant and hypervirulent isolates through plasmid recombination and fusion events. Consequently, carbapenem-resistant *K. pneumoniae* (CRKP) and hypervirulent *K. pneumoniae* (hvKP) have merged (Yang et al., 2021). The carriage of a virulence plasmid containing two capsular polysaccharide (CPS) regulator genes (*rmpA* and *rmpA2*) and several siderophore gene clusters (*iucABCD*/*iutA*/*iroBCDN* clusters) is thought to contribute to the hypermucoviscous phenotype of hvKP (Russo et al., 2018; Russo and Marr, 2019). Globally, there has been a growing incidence of MDR and hvKP isolates carrying the *bla*_OXA-48_, *bla*_OXA-181_, and *bla*_OXA-232_ genes (Pitout et al., 2019), likely resulting from two converging evolutionary pathways of *K. pneumoniae* involving plasmid recombination and fusion events (Zhang et al., 2020; Yang et al., 2021). We report the emergence of hvKP isolates carrying both *bla*_OXA-232_ and *bla*_OXA-181_ recovered from patients at a teaching hospital in southern China. The high ICU occupancy (62%) and mortality rates (52%) of patients infected with these bacteria highlight the importance of monitoring this isolate. In recent years, an increasing number of *K. pneumoniae* isolates with integrated hypervirulence and carbapenem resistance phenotypes have been identified, resulting in devastating clinical outcomes (Yang et al., 2022). Our research team found that OXA-resistant isolates with hypervirulence had begun to prevail in China during our active surveillance of CRKP isolates.

In our study, all *bla*_OXA-48-like_ positive CR-hvKP isolates exhibited resistance to commonly used clinical antimicrobial agents and harbored at least one virulence gene located on a pLVPK-like virulence plasmid, including *iroB*, *iucA*, *peg-344*, *rmpA*, and *rmpA2* genes. Although OXA-48-like represents weak activity of carbapenemase (Oueslati et al., 2015), all these isolates demonstrated high resistance to carbapenem antimicrobial agents such as imipenem (100%), meropenem (94%), and ertapenem (90%), consistent with the results of another study (Jia et al., 2021). In addition, we detected at least one extended-spectrum β-lactamase (ESBL) gene, such as *bla*_CTX-M_, *bla*_SHV_, or *bla*_TEM_, which might contribute to the observed drug-resistant phenotype.

Horizontal transmission of resistance genes via mobile plasmids is a common dissemination mechanism for carbapenemase-producing Enterobacteriaceae (CPE), resulting in rapid spread of resistance genes across diverse isolates and hosts (Pulss et al., 2018). The high proportion of ST11 (30/50) among the *bla*_OXA-48-like_ positive CR-hvKP isolates in our study suggests common clonal origins (Figure 2). Our study further revealed that three clusters of isolates (A, E, and P) were closely related (Figure 2), with PFGE patterns and MLST demonstrating both clonal and plasmid transmission. These findings suggest that horizontal gene transfer/plasmid transfer plays a crucial role in the dissemination of CR-hvKP strains. Notably, we found a high prevalence of triple positivity for multiple carbapenemases in eight isolates (five producing *bla*_KPC + NDM + OXA-181_ and three producing *bla*_KPC + NDM + OXA-232_). Our results are consistent with the continuous global emergence of multidrug-resistant strains (Hu et al., 2014; Khan et al., 2019), which can be sustained by diverse mechanisms, such as R plasmids or transposons (Miller et al., 2014).

In our study, we identified three plasmid replicons (ColE, IncF, and IncX3) with high frequency in our isolates. Previous research has demonstrated that the *bla*_NDM_ gene can be found in various types of plasmids, including those in the IncF, IncFII, IncN, and IncX3 incompatibility groups (Zhu et al., 2016). The initial acquisition of the OXA-181 gene occurred through the mediation of ISEcp1, which subsequently integrated into Tn2013 and was found on several plasmids such as ColE2, IncX3, IncN1, and IncT. On the other hand, the genetic environment surrounding *bla*_OXA-232_ is very similar to that of *bla*_OXA-181_, with the former differing from the latter by only one amino acid substitution (Pitout et al., 2019). Plasmids harboring *bla*_KPC_ genes, ranging in size from 10 to 300 kb, are commonly found in various incompatibility groups, such as IncF, IncI, IncA/C, IncN, IncX, IncR, IncP, IncU, IncW, IncL/M, and ColE (Chen et al., 2014). In our plasmid conjugation experiment, eight isolates carrying three carbapenemase resistance genes were successfully conjugated. However, Potron A. and colleagues discovered that the plasmid-mediated carbapenem-resistance gene *bla*_OXA-232_ was located on a small, non-conjugative plasmid called pOXA-232, which carried a ColE-type backbone (Potron et al., 2013; Abdul Momin et al., 2017; Weng et al., 2020). Our research team discovered for the first time the emergence of super resistant bacteria due to the lack of reports on *bla*_KPC + NMD + OXA_ resistant strains of super carbapenem. Our next challenge is to investigate how these three resistant plasmids can facilitate transfer *in vivo*, as well as the mechanism underlying their coexistence.

pLVPK-like virulent plasmids often have a strong correlation with high hypervirulent phenotypes in *K. pneumoniae*. Several experiments to confirm the virulent phenotype of these *bla*_OXA-48-like_ positive CR-hvKP isolates: hypermucoviscosity (String Test), serum killing assay (*in vitro*) and *Galleria mellonella* infection model (*in vivo*), these isolates have been shown to be hypervirulent. We performed the localization of virulence plasmids for eight strains that carried three resistance plasmids at the same time. Southern blotting hybridization determined that these CRKP carried a pLVPK-like virulent plasmid (138.9–216.9 kb) with uneven numbers and sizes of plasmids. These findings suggest the emergence of hv-CRKP isolates that simultaneously carry three carbapenemases and a virulence plasmid. We speculate that our hv-CRKP isolate may have acquired a virulence plasmid during the evolution of the drug-resistant isolate.

Given these findings, it is essential to carefully monitor and conduct follow-up studies to gain further insights into the epidemiology of multidrug-resistant strains, as well as the possible evolution of successful plasmids and transposition modules that contain three antimicrobial resistance genes (*bla*_OXA-48-like + NDM + KPC_) of clinical relevance. Our active surveillance of CRKP isolates led to the discovery of OXA-resistant isolates with hypervirulence that have become prevalent in China. These hypervirulent OXA-resistant isolates carry a pLVPK-like virulence plasmid containing the *iroB*, *iucA*, *peg-344*, *rmpA*, and *rmpA2* genes. Furthermore, we found eight clinical isolates of hv-CRKP carrying three carbapenem-resistant genes: *bla*_KPC_, *bla*_OXA-181 or OXA-232_, and *bla*_NDM-1_. This is the first report of the co-production of three carbapenemase genes (*bla*_KPC + NDM + OXA181_ and *bla*_KPC + NDM + OXA232_) in CRKP isolates, highlighting the need for active surveillance to control further transmission.

## 5. Conclusion

This study reports the emergence of hypervirulent OXA-48-like-producing hv-CRKP in our hospital. Based on PFGE and MLST results, two genetic relationships were identified: clonal and plasmid transmission. The most common OXA-like carbapenemases were *bla*_OXA-181_ and *bla*_OXA-232_, which predominantly clustered in K64 ST11 and K47 ST15 isolates. Notably, we identified the co-production of three carbapenemases genes (*bla*_KPC + NDM + OXA181_ or _OXA232_) and a pLVPK-like virulence plasmid in hv-CRKP isolates, which to our knowledge, has not been previously reported. These hv-CRKP isolates carried a pLVPK-like virulence plasmid ranging from 138.9 to 216.9 kb. Therefore, implementation of effective infection control measures is urgently needed to prevent further spread in the region.

## Data availability statement

The original contributions presented in the study are included in the article/supplementary material, further inquiries can be directed to the corresponding authors.

## Author contributions

The study was designed by WZ and YL, while T-xP, W-yL, and PL conducted the experiments. SL, Z-yH, and D-DW carried out the analysis. T-XX, PL, and W-JL drafted the manuscript. All authors contributed to the article and approved the submitted version.

## Funding

This research was funded by the National Natural Youth Science Foundation of China (grant number 82102411 and 82200194); The Natural Science Key Project of Jiangxi Province (grant numbers 20202ACBL206025 and 20202ACBL206023); the Project of Science and Technology Innovation Talents in Jiangxi (grant number JSXQ2019201102); Jiangxi Provincial Department of Science and Technology (grant number 20202ZDB01016); the Science and technology plan of Jiangxi Health Committee (grant number SKJP-220212554); the Clinical Research Nurture Project of the First Affiliated Hospital of Nanchang University (grant number YFYLCYJPY202001); and Youth Scientific Research Foundation of the First Affiliated Hospital of Nanchang University (grant number YFYPY202115); and the Science and Technology Plan of Jiangxi Provincial Health Commission (202130137), the Natural Science Foundation of Jiangxi province (grant number 20224BAB216084), The first affiliated hospital of Nanchang University Young Talents Scientific Research Breeding Fund (grant number YFYPY202114) and Science and Technology Research Project of Jiangxi Provincial Department of Education (grant number GJJ2001448).

## Conflict of interest

The authors declare that the research was conducted in the absence of any commercial or financial relationships that could be construed as a potential conflict of interest.

## Publisher’s note

All claims expressed in this article are solely those of the authors and do not necessarily represent those of their affiliated organizations, or those of the publisher, the editors and the reviewers. Any product that may be evaluated in this article, or claim that may be made by its manufacturer, is not guaranteed or endorsed by the publisher.

## Footnotes

^1^ http://www.eucast.org/clinical_breakpoints/
